# Identification of the Destruction Model of Ventilated Facade under the Influence of Fire

**DOI:** 10.3390/ma13102387

**Published:** 2020-05-22

**Authors:** Krzysztof Schabowicz, Paweł Sulik, Łukasz Zawiślak

**Affiliations:** 1 Faculty of Civil Engineering, Wrocław University of Science and Technology, Wybrzeże Wyspiańskiego 27, 50-370 Wrocław, Poland; krzysztof.schabowicz@pwr.edu.pl; 2 Instytut Techniki Budowlanej, Filtrowa 1, 00-611 Warszawa, Poland; p.sulik@itb.pl

**Keywords:** ventilated facades, large-scale facade model, fire safety, fiber cement board, large-slab ceramic tile

## Abstract

Ventilated facades are becoming an increasingly popular solution for external part of walls in the buildings. They may differ in many elements, among others things: claddings (fiber cement boards, HPL plates, large-slab ceramic tiles, ACM panels, stone cladding), types of substructures, console supports, etc. The main part that characterizes ventilated facades is the use of an air cavity between the cladding and thermal insulation. Unfortunately, in some aspects they are not yet well-standardized and tested. Above all, the requirements for the falling-off of elements from ventilated facades during a fire are not precisely defined by, among other things, the lack of clearly specified requirements and testing. This is undoubtedly a major problem, as it significantly affects the safety of evacuation during a fire emergency. For the purposes of this article, experimental tests were carried out on a large-scale facade model, with two types of external-facade cladding. The materials used as external cladding were fiber cement boards and large-slab ceramic tiles. The model of large-scale test was 3.95 m × 3.95 m, the burning gas released from the burner was used as the source of fire. The test lasted one hour. The facade model was equipped with thermocouples. The cladding materials showed different behavior during the test. Large-slab ceramic tiles seemed to be a safer form of external cladding for ventilated facades. Unfortunately, they were destroyed much faster, for about 6 min. Large-slab ceramic tiles were destroyed within the first dozen or so minutes, then their destruction did not proceed or was minimal. In the case of fiber cement boards, the destruction started from the eleventh minute and increased until the end of the test. The authors referred the results of large-scale test to testing on samples carried out by other authors. The results presented the convergence of large-scale test with samples. External claddings was equipped with additional mechanical protection. The use of additional mechanical protection to maintain external cladding elements increases their safety but does not completely eliminate the problem of the falling-off of parts of the facade. As research on fiber cement boards and large-slab ceramic tiles presented, these claddings were a major hazard due to fall-off from facade.

## 1. Introduction

Ventilated facades are a type of external part of multilayer wall, which has a construction part and is usually a masonry or concrete wall, but it can also be a wooden or steel structure. The wall is then fitted with insulation, with consoles holding the elements of the substructure and the external cladding. This external cladding protects the aforementioned layers against environmental influences and gives the final shape and appearance of the facade. There is an air gap between the external cladding and the insulation, also known as a ventilation gap. The width of the air gap in ventilated facades ranges from 20 to 50 mm [1,2], some sources also provide higher values, e.g., from 40 to 100 mm [3]. Ventilated facades allow for shaping external claddings from various materials, structures, textures or colors. Due to their high aesthetics, ventilated facades are increasingly often used as external parts of newly built buildings, but they are also perfect for buildings undergoing renovations. External cladding elements can have very large individual elements. The standard dimension for fiber cement boards is 1250 mm × 3100 mm [1] and for HPL 1850 mm × 4100 mm [1].

Regulations control a number of requirements for external walls, such as ensuring appropriate thermal insulation, the requirements for durability and protection of the building according to [4,5] and safety of use in environmental and emergency situations. One of the most important requirements a building must meet in emergency situations, is the impact of fire, is to ensure the possibility of evacuation of users and work of rescue teams [5]. External walls with external facade cladding must ensure, among other things, sufficient durability in emergencies, i.e., prevent facade elements from falling off. This problem is widely known throughout Europe.

The European Commission in [5], presented analysis of today’s requirements of the falling-off of parts of facades in countries. In [5] standardization proposals were also presented, among others of falling-off cladding elements during a fire. Propose two methods for assessing falling elements of the façade, the first one was dependent on fire class:

- No falling-off of parts larger than 1 kg and 0.1 m^2^ (class F1);
- No falling-off of parts larger than 5 kg and 0.4 m^2^ (class F2);
- No burning particles at all (class D0);
- Limited duration burning debris <20 s (class D1).

There was presented also alternative test method: falling parts are limited to a maximum of 1 kg and an area of 0.1 m^2^ for each individual piece. Until the standards are harmonized in Europe, manufacturers of entire ventilated façade systems, designers and contractors [6,7,8] will have a problem delivering these products within the European Union.

There are many standards in the world for the large-scale facade test [9,10,11,12]. They are based on the spread of fire from a recess/hole, simulating the window openings of a room in a real building. The fire source is located there, defined by the normal temperature action curve. The flames escape from the recess affecting the external facade cladding and other wall elements. Individual standards differ in details, i.e., the type of fire source: wood crib [9,10,11] or propane-butane gas [12], dimensions of the recess/hole, test time, shape of the facade model in large scale and its dimensions. The comparison of individual standards for the test of large—scale facade models in the scope of fire safety is presented by Smolka in [13]. Due to the growing awareness of the phenomenon of fire spreading on the external part of the elevation and a number of threats caused by this phenomenon, the European Commission started an attempt to harmonize the testing standards [11].

Sulik and Kinowski [14,15] analyzed the large-scale facade models at the impact of fire. Ventilated facades with different external claddings have been assumed as initial conditions. The external claddings include fiber cement boards and high-pressure laminate (HPL) panels, ceramic tiles, natural stone and synthetic stone marmoglass (glass conglomerate), layered steel ACM panels (aluminum composite material). During the research, a number of dependencies were noted, among others: that the way the external facade cladding is installed has an impact on its safety. Mechanical assembly is safer than adhesive assembly. The requirements for the test duration are usually 60–120 min. After 30 min the destruction of the cladding is minimized or even stopped in some cases. The claddings that have been positively tested in this type of test are fiber cement boards and ACM panels, in which case the falling-off of parts were up to 2 kg. In the case of ceramic tiles, the falling-off of parts were also of acceptable weight, but they were sharp and posed a risk to evacuating people. In the case of stone cladding and marmoglass, the weight of the falling elements was several or even several dozen kilograms. Sulik and Kinowski [6] also presents the verification of fire safety of glass facades. The results indicate a problem with the falling-off of elements of facades.

It should also be emphasized that fiber cement boards are not well identified when it comes to the conditions of fire and high temperatures. Szymków’s research presented in the study [16] showed that fibers in fiber cement boards are destroyed at 230 °C only after 3 h. Destruction of such boards during the bending test takes place through brittle high-energy cracking. Other fiber-reinforced materials show similar behavior. The study [17] shows that the decrease of compression strength of concrete and fiber-reinforced concrete at temperatures up to 300 °C is about 10%. On the other hand, in the case of fiber-reinforced cement composites, the modulus of rupture increases with the temperature increase up to about 300 °C [18]. Temperatures up to about 300 °C for fiber-reinforced materials are relatively safe in a short period of time. Their destruction takes place only after a longer period of time, usually after several hours. Szymków in his work [16] also carried out tests on samples of fiber cement boards at 400 °C. At this temperature, the samples showed much lower stability and were destroyed much faster. The results had large discrepancies because, depending on the manufacturer, components, manufacturing technology, they “withstood” for up to several minutes. Some were destroyed even in a shorter period of time. Unfortunately, in case of fire, the temperature impact on external facade claddings may reach a value locally even up to 900 °C (the external curve provides a value of 660 °C), and such experimental tests are not available for fiber cement boards. Looking at the above analogies of other materials with the use of fibers, interesting conclusions are contained in the study [17], where the tests for concrete and fiber-reinforced concrete were performed. It was found that the temperature of 800 °C reduces the compressive strength of concrete and fiber-reinforced concrete class C30/37 by over 90%. In the case of high-quality concrete and fiber-reinforced concrete of class C60/75, the compression strength decreases by more than 90%, only after the samples are heated at 1000 °C. At 500 and 600 °C, the samples without the addition of fibers were destroyed during their annealing, whereas those with the addition of polypropylene fibers retained their residual bending strength [18]. The study [19] also showed a positive effect of using fibers to increase the bending strength of beams subjected to the normative fire temperature curve. The fibers have a positive effect on increasing the load capacity of the elements under the influence of high temperatures, e.g., in an emergency situation, such as a fire.

The authors of this study and [20,21,22,23,24,25,26], due to the lack of scientific literature on the problem of destruction of fiber cement boards used as external facade claddings in ventilated facades, have attempted to analyze this issue. This analysis was based on the large-scale model of facade. This issue is important because the popularity and demand for ventilated facades is increasing, and unfortunately the problem quoted by the authors concerns the safety of these facades in an exceptional situation, i.e., the impact of fire.

To sum up, it can be argued that today practically no type of material used for external facade claddings (except for steel sheets) ensures that the condition specified in the Regulation [5] is met. It is therefore necessary to apply some kind of compromises and, above all, to harmonize the testing standards and analysis of these results.

## 2. Materials and Model of Ventilated Facade

In order to solve the scientific task, a model was prepared to reproduce the facade of the building, made in the so-called large-scale model. The model was constructed with reference to the existing wooden frame building systems.

The analyzed wall was a part of the wooden panel and modular skeleton construction system. The construction of the elements consisted of a wooden skeleton with a glass wool insulation material filling.

**Figure 1:**
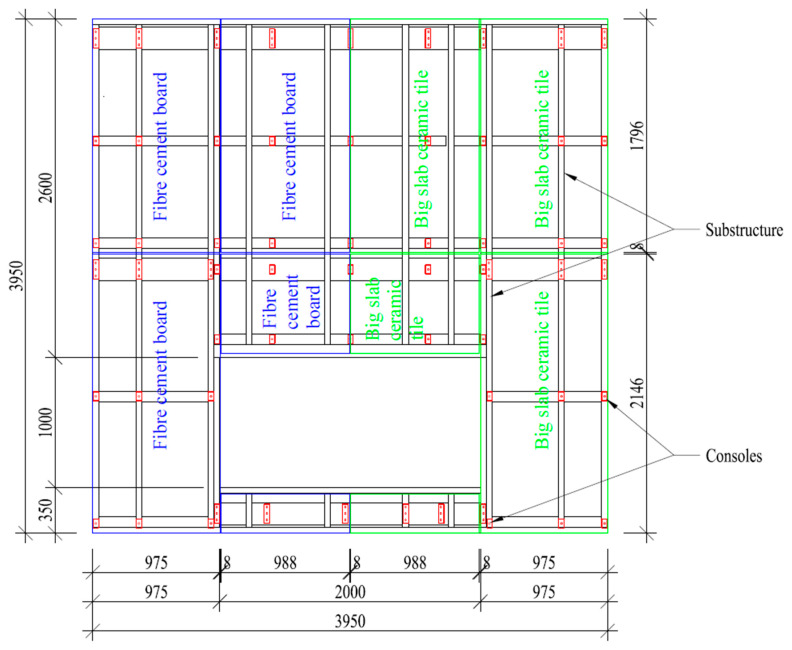
Scheme of the aluminum substructure, console arrangement, external cladding panels arrangement and material indication.

The subject of the research verification was the layout of the ventilated facade, which included two variants of external claddings: fiber cement boards and large-slab ceramic tiles. The external cladding was attached to steel consoles. The supporting structure was a wooden. Between the posts there was a layer of thermal insulation made of glass wool. On the inside and outside of the wall there was a layer of 12.5 mm plasterboard. The substructure fastening the external cladding was made of an aluminum grate with a section of L60 × 40 mm × 2 mm, screwed to steel consoles. The consoles were fixed to the system skeleton wall (model’s supporting element) by means of 8 × 60 steel disc screws. The layout of the aluminum substructure and the consoles transferring loads from the aluminum substructure to the system skeleton wall is shown in Figure 1.

The external cladding of the ventilated facade consisted of fiber cement boards with a thickness of 8 mm and density of 1700 kg/m³ and large-slab ceramic tiles with a thickness of 5.6 mm and density of 2855 kg/m³. The external facade claddings attached to the aluminum grate were made with the use of the system adhesive technology and additionally with the use of steel mechanical connectors, i.e., perforated steel tapes attached to the substructure. The dilatation between individual boards was 8 mm. Figure 1 shows the division scheme of the external cladding and indicates the material of which they were made, namely, the fiber cement boards in the left part and the large-slab ceramic tiles in the right part.

**Figure 2:**
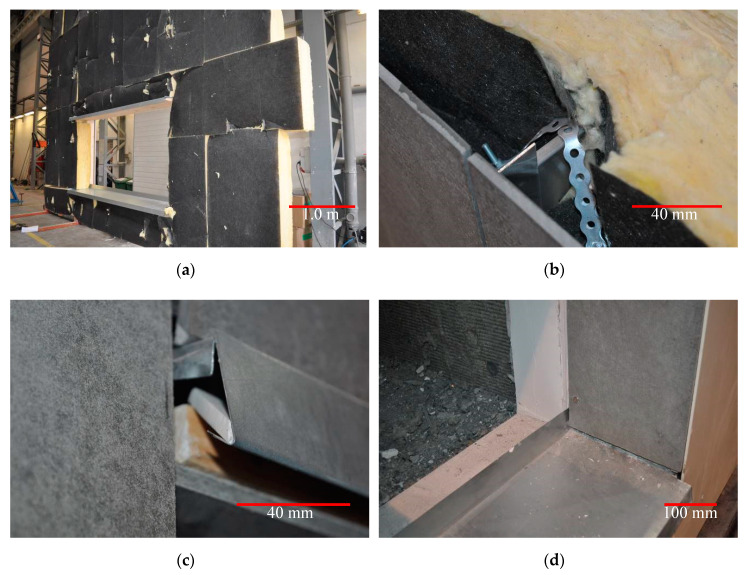
Selected details of the ventilated facade model under test: (**a**) thermal insulation system; (**b**) the detail of perforated tape connection with the console; (**c**) the detail of closing sheet above the window opening; (**d**) the detail of sill system.

Selected details of the tested ventilated facade are shown in Figure 2. Total dimensions of the tested model of ventilated facade were 3950 mm × 3950 mm, air gap width 38 mm, dimensions of fire hole 2000 mm × 1000 mm.

**Figure 3:**
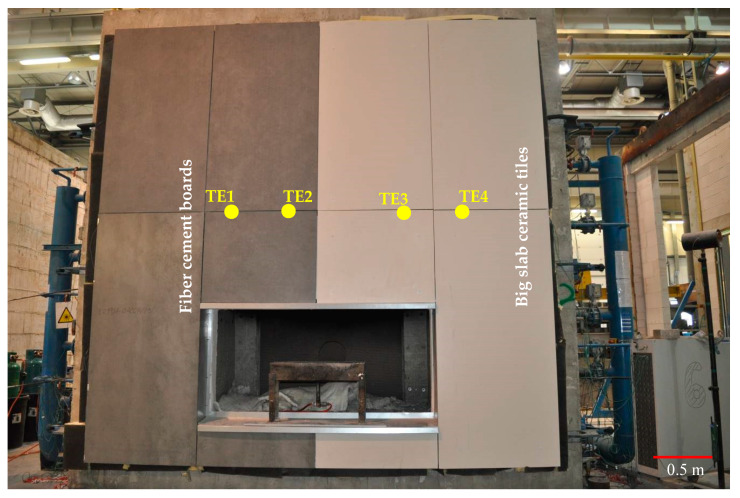
Ventilated facade model with location of thermocouples.

The scenario of the ventilated facade fire assumed that the flames would escape through a window opening from a room located directly behind the facade, inside the building. In order to map the room from which the flame was emitted, a recess was made in the model of the facade where the source of fire was placed. The burner parameters were selected in such a way as to reproduce the standard fire in the room, defined in the fire resistance test standard [20]. The fire was mapped with a gas burner releasing propane-butane gas at the speed of 3.8 m/s. The air from the recess in which the so-called sand burner was placed, supplied by the furnace installation had the possibility of inflow from the side of the opening, through a technical solution ensuring laminar air inflow, the so-called vent. In order to verify and identify damage to the external cladding, four thermocouples were installed. Thermocouples send information regarding temperatures as a function in time. Influence of high temperature was one of the negative external environmental effects on buildings elements. Thermocouples were placed in the gaps between the panels: two in the part of fiber cement boards and two in the part of large-slab ceramic tiles. The location of the thermocouples is shown in Figure 3. A ventilated facade with open joints was made, where additional gaps (gaps between individual external cladding panels) can provide air circulation.

## 3. The Test and Results

**Figure 4:**
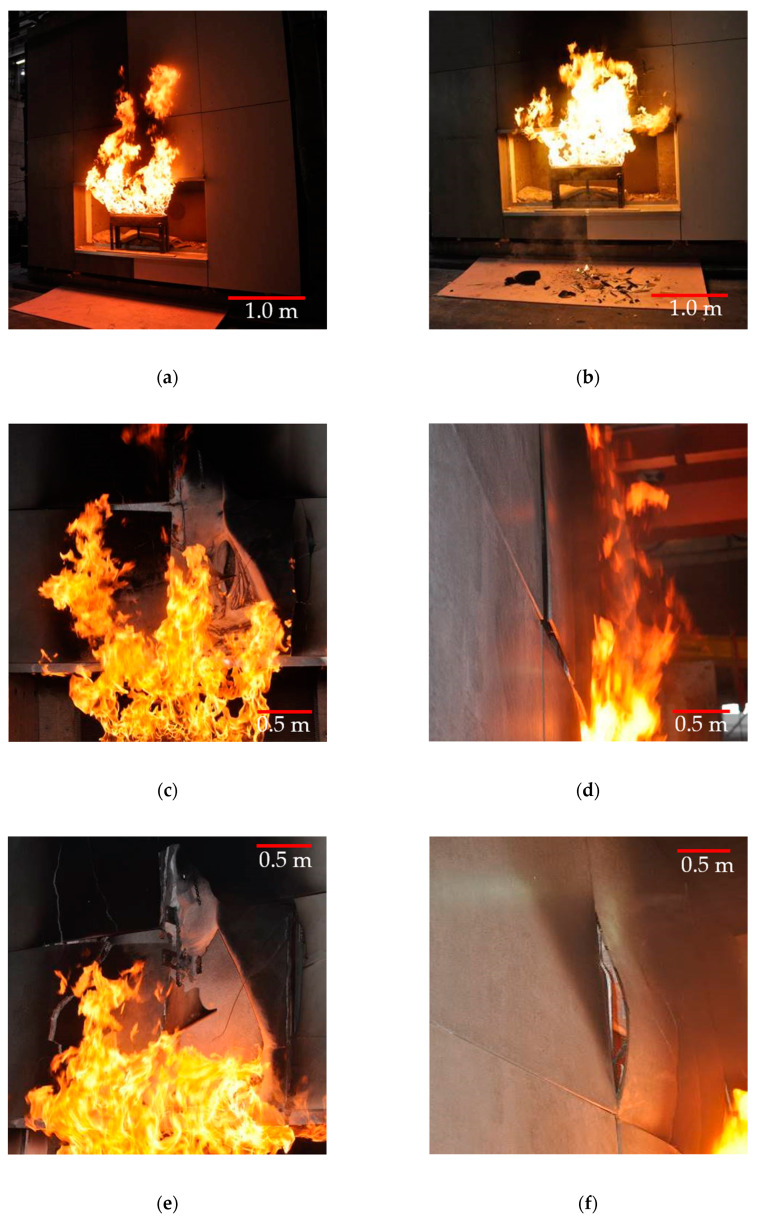

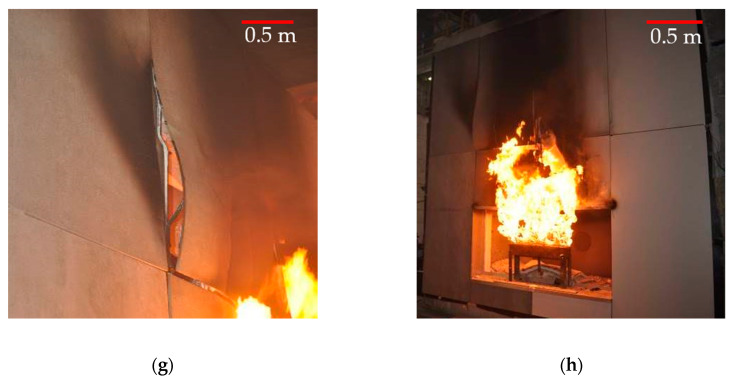
Ventilated facade model view in subsequent test minutes.: (**a**) 1st minute of the test; (**b**) 6th minute of the test; (**c**) 11th minute of the test; (**d**) 21st minute of the test; (**e**) 31st minute of the test; (**f**) 41st minute of the test; (**g**) 56th minute of the test; (**h**) 61st minute of the test.

The test was carried out in a closed hall, with an ambient temperature of 18.9 °C and relative humidity of 60.7%. The test started with setting the burner and calibrating the gas release. The first 5 min of the fire revealed smoke/charring of the external cladding, no falling-off of the external cladding elements (Figure 4a).

The first falling-off of elements were observed in the 6th minute of the test, i.e., large-slab ceramic tiles elements started to fall off (Figure 4b). The subsequent minutes of high temperature impact caused greater destruction of the external cladding, particularly visible in the part where large-slab ceramic tiles were located. On 11th minute, the degradation of fiber cement boards started significantly. Figure 4c shows the initial stages of expansion of the fiber cement boards. The destruction of the large-slab ceramic tiles slowed down around 20th minute. The places where the temperature impact was highest were destroyed, and in the remaining places, large-slab ceramic tiles “tolerated” high temperatures quite well. Large-slab ceramic tiles had the greatest degradation in the first several minutes of fire. No further degradation of ceramic sinters was noticed.

These materials destruct themselves also in another way. In the case of large-slab ceramic tiles, which have a uniform structure, the destruction takes place by cracking and falling-off of pieces of elements. In the case of fiber cement boards and their nonuniform structure, the boards, despite significant warping (deplanation) caused by high temperature (e.g., Figure 4e), do not fall off among others due to the good tensile properties of the fibers. Only complete destruction of the fibers causes the elements to fall off.

The test revealed cracking and falling-off of fragments of both fiber cement boards and large-slab ceramic tiles. This mainly concerned the external cladding located above the opening, i.e., above the fire source. Some of the claddings have detached from the grate but have not fallen off and hang on perforated steel tapes mechanically attached to the cladding. The maximum mass of a single component which fell off and dropped during the test was 1.15 kg. This was due to a safety system using a steel perforated tape and mechanical fasteners. Degradation of the large-slab ceramic tiles took place during the first few minutes, then this part of the facade was stable. Fiber cement boards behaved differently. The first minutes showed the stability of the fiber cement boards. From 11th minute, the boards started to show significant degradation progressing practically until the end of the test. If perforated steel tapes were not used, the cladding elements falling off the facade would probably be of considerable dimensions.

**Figure 5:**
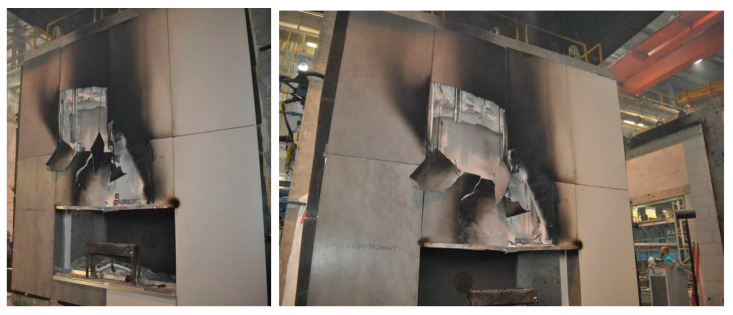
Ventilated facade model view after the fire source was extinguished and the test was completed.

The horizontal upper splay, made of 0.5 mm thick steel sheet, deformed, but its location has not changed. The aluminum grate directly above the recess with the fire source, except in places directly sheltered by the steel top splay was burnt at a height of up to 1660 mm. Glass mineral wool was melted directly above the recess at a height of up to 1400 mm. The steel fasteners and consoles remained in place, as did the lower splay. Figure 5 shows the condition of the facade after the fire source was extinguished.

**Figure 6:**
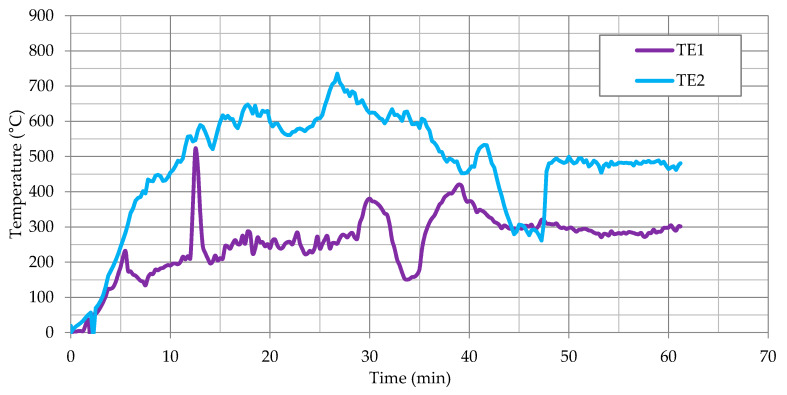
Temperature measurement results for TE1 and TE2 thermocouples.

**Figure 7:**
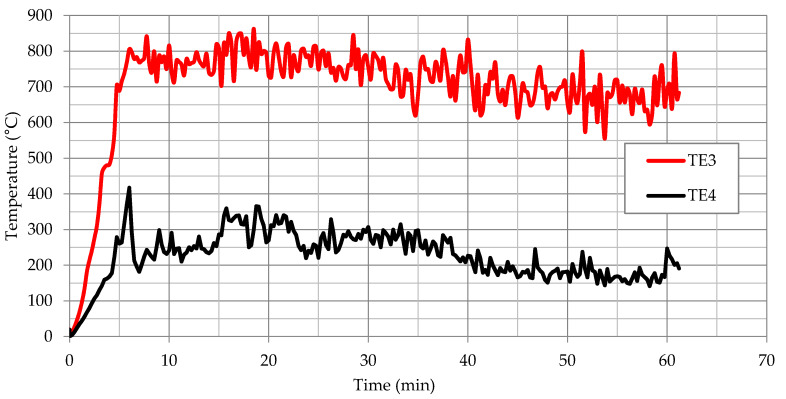
Temperature measurement results for TE3 and TE4 thermocouples.

The results of temperature measurement from the thermocouples located in the part of the fiber cement boards, as shown in Figure 3, are presented as a function of time in Figure 6 for thermocouples TE1 and TE2. On the other hand, the results for TE3 and TE4 thermocouples located in the part of large-slab ceramic tiles are shown in Figure 7. The results presented for TE1 and TE2 thermocouples show high instability and temperature variations. This was due to the high development of degradation over time of fiber cement boards, especially after full development of the fire. The locations of the greatest temperature fluctuations in Figure 6 can be associated with cracking or detachment of external cladding elements.

Thermocouples TE3 and TE4, as opposed to thermocouples located in a part of fiber cement boards, initially showed higher temperature increase. The power of the fire was constant, the difference in temperature was caused by the heating of the elements. In 6–7 min, TE3 thermocouples showed high stability—large-slab ceramic tiles were quickly destroyed in the central part above the fire recess. TE4 thermocouple initially showed high instability and temperature fluctuations. This was caused by the influence of lower temperature on large-slab ceramic tiles in this part of the facade. TE4 thermocouple became stable in about 15th minute when the ceramic sinters were no longer damaged by high temperature. The damage is shown in Figure 4c. This state or a slightly altered state, was maintained practically until the end of the test.

## 4. Discussion

The test of a model of a ventilated facade made of two different external claddings showed significant material differences. Large-slab ceramic tiles very quickly, in the first few minutes or so, were destroyed by high temperatures, then their degradation does not deepen. The course of events in the part made of fiber cement boards looks different. In the initial stage of the fire, the panels show high resistance—the first few minutes or so. About 11 min, the first signs of degradation of fiber cement boards are visible (Figure 4c). Then the state of the degradation was deepened until the final destruction. Fiber cement boards have exhausted their load-bearing capacity during high temperature exposure. They were held on the structure only by perforated steel tapes. The authors wanting to refer the results from large-scale test to testing on samples by Szymków [1], had to specify the method of reference of the results. The temperature in the large-scale test was much higher than in Szymków’s research [1], the time function will be not representative. The authors determined that as the most reflecting value will be function of integral.

The integral corresponding to the temperature function from time was determined for all thermocouples. The following results were obtained:

- the integral for TE1 (°C × min) was 15,745.0 (°C × min);
- the integral for TE2 (°C × min) was 29,475.1 (°C × min);
- the integral for TE3 (°C × min) was 42,686.2 (°C × min);
- the integral for TE4 (°C × min) was 13,912.0 (°C × min).

**Figure 8:**
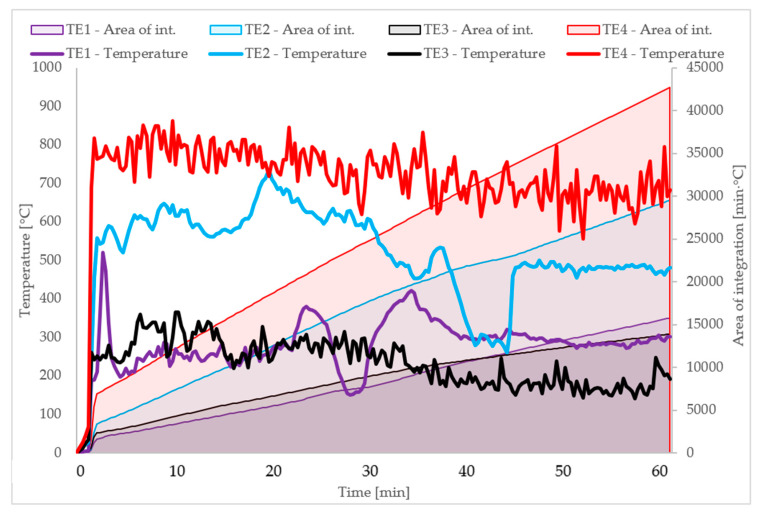
Temperature measurement results for thermocouples and the increasing integral for the temperature-time function.

These results are shown in Figure 8 together with temperature diagrams for all thermocouples: TE1, TE2, TE3, TE4. In this graph we can observe a linear growth pattern of the integral for TE3 and TE4 thermocouples (thermocouples placed in a part of the cladding made of large-slab ceramic tiles). In addition, in the part with ceramic sinters the fire force was much higher. The maximum temperature for TE3 was 862.7 °C and for TE2 thermocouple measuring the temperature in the area of fiber cement boards it was 735.5 °C. The much higher temperatures in the large-slab ceramic tiles area were caused by the much faster destruction of this material, the “release” of access to the thermocouples. The falling-off of large-slab ceramic tiles made easier way to burn mineral wool, this allows maintain higher temperature. In addition, it was likely that ceramic sinters insulate thermocouples much worse than fiber cement boards. The temperature course for TE1 and TE2 thermocouples contains much more disturbances and significant faults. In addition, the integral function has a visible refraction in 34th and 39th minutes for TE1 thermocouple and 42nd and 48th minutes for TE2 thermocouple.

Szymków in [16] carried out tests to identify the degree of destruction of fiber cement boards under high temperatures. He analyzed the influence of temperature 400 °C in a given unit of time. Different samples of 20 mm × 100 mm fiber cement boards were subjected to high temperature influence in 1 to 15 min. The samples differed in technical parameters, composition, production technology and area of application and were tested at different times. Characteristic parameters of individual sample series given by the manufacturer are presented in Table 1.

For the purposes of the articles, selected test results from [16] were used. Fracture energy *W_f_* and modulus of rupture *MOR* were analyzed. The results are presented in Table 2.

As the results shown in the study [16] show, for fiber cement boards at high temperature of 400 °C, the fracture energy increased in the initial phase. In case of B, C, D series boards, the fracture energy increased from 2% to 9%. Then a decrease in the fracture energy was visible for all types of fiber cement boards. The same was true for modulus of rupture *MOR*, which increased from 3% to 20% in the initial period and then decreases. Table 3 shows the aggregate summary of the temperature function integral from the maximum time that a sample could be tested for the A–E series of boards.

For the purpose of further analyses, the average value of the function integral for all series was assumed to be 3800 (°C × min). The average value of the function integral for all series corresponds to the increasing integral for TE1 thermocouple in about 20.5 min and the increasing integral for TE2 thermocouple in about 12.75 min. This is reflected in Figure 6; Figure 8. The time of 12.75 min approximately coincides with the point of significant fault in the temperature graph for TE1 thermocouple. This corresponds to the beginning of the destruction in 11th minute of the tested model (Figure 5). Fiber cement boards detach into parts, probably obscuring the TE1 thermocouple. Then the fiber cement panels were continuously destroyed until the end of the test. This corresponds to integral 42,686.2 (°C × min). The tests shown in [16] end with the integral value of 6000 (°C × min). The results obtained during the test in question indicate identical tendencies to the behavior of the samples presented in [16].

## 5. Conclusions

The model of large-scale ventilated facade is a huge source of knowledge about its behavior during a fire. The problem of destruction of the external cladding in the case of fiber cement boards and large-slab ceramic tiles has not been sufficiently recognized so far and such studies as presented in the article indicate trends in the “behavior” of the facade and these claddings.

Fiber cement boards pose a great threat to the safety of use in case of flames escaping from window openings to facades during a fire. Large-slab ceramic tiles appear to be a safer form of external cladding for ventilated facades. Unfortunately, they were destroyed much quicker, i.e., starting from the 6th minute. The danger of falling elements passes after a dozen or so minutes of fire. In the case of fiber cement boards, the visible destruction starts from about 11th minute and runs throughout the whole period of high temperature impact. Falling-off of elements in the case of fiber cement boards were large sizes, even despite the use of additional protections. In the case of standard mechanical or adhesive fastening, fiber cement boards would pose an even greater threat.

In the next part of the article compared behavior of fiber cement boards on samples test with the large-scale facade test. The temperature integral was taken as the comparative value of the samples with the large-scale façade test. The results of both tests show convergent results. The samples were a good alternative initial verification of the facade cladding behavior in fire conditions in global analysis.

The authors are planning next research in this field, developing the model and using more thermocouples. The tests will be carried out on a greater number of different types of claddings.

The next research steps, helping to better solve the problems of ventilated facades and to increase their safety, should, according to the authors, concern the samples of fiber cement panels tested at a temperature closer to the actual fire, i.e., about 550–650 °C (Figure 6). Such tests should also be carried out for ceramic sinters, which have not yet been described in the literature.

## Figures and Tables

**Table 1:** Characteristic parameters of individual sample series.

Boards Series Designation	Board Thickness (mm)	Board Color	Application	Bulk Density (g/cm^3^)	Pressing During Production	Modulus of Rupture *MOR* (MPa)
SERIE A	8.0	Natural color	External	1.60	Yes	25
SERIE B	341.97	Dyed in the mass	External	1.60	Yes	30
SERIE C	139.37	Dyed in the mass	External	1.65	Yes	30
SERIE D	133.65	Natural color	External	1.70	Yes	20
SERIE E	281.84	Natural color	external	1.20	Partially	12

**Table 2:** Aggregate summary of averaged values of fracture energy *W_f_* and modulus of rupture MOR for panels, under the influence of high temperature 400 °C from 1 to 15 min [1].

Boards Series Designation	Fracture Energy *W_f_* (J/m^2^)	Modulus of Rupture *MOR* (MPa)
SERIE A2—1 min of influence	372.82	20.89
SERIE A3—2.5 min of influence	341.97	23.05
SERIE A4—5 min of influence	139.37	17.68
SERIE A5—7.5 min of influence	133.65	15.50
SERIE B2—1 min of influence	281.84	23.30
SERIE B3—2.5 min of influence	302.99	23.80
SERIE B4—5 min of influence	301.84	25.56
SERIE B5—7.5 min of influence	309.52	30.29
SERIE B6—10 min of influence	229.02	23.22
SERIE B7—12.5 min of influence	134.53	14.13
SERIE B8—15 min of influence	86.94	12.53
SERIE C2—1 min of influence	1375.14	35.75
SERIE C3—2.5 min of influence	1396.19	44.61
SERIE C4—5 min of influence	320.86	22.44
SERIE C5—7.5 min of influence	276.12	17.63
SERIE C6—10 min of influence	30.67	7.98
SERIE D2—1 min of influence	297.11	23.27
SERIE D3—2.5 min of influence	298.18	26.36
SERIE D4—5 min of influence	233.90	23.90
SERIE D5—7.5 min of influence	204.31	21.31
SERIE D6—10 min of influence	152.94	15.31
SERIE E2—1 min of influence	736.44	14.41
SERIE E3—2.5 min of influence	555.62	11.89
SERIE E4—5 min of influence	250.92	10.11

**Table 3:** Aggregate summary of the temperature function integral from the maximum time [1].

Boards Series Designation	Fracture Energy *W_f_* (J/m^2^)	Modulus of Rupture *MOR* (MPa)	Function Integral (°C × min)
SERIES A5—7.5 min of influence	133.65	15.50	3000
SERIES B8—15 min of influence	86.94	12.53	6000
SERIES C6—10 min of influence	30.67	7.98	4000
SERIES D6—10 min of influence	152.94	15.31	4000
SERIES E4—5 min of influence	250.92	10.11	2000
